# Laparoscopic splenectomy for polysplenia with splenic torsion: a case report

**DOI:** 10.1186/s40792-019-0582-0

**Published:** 2019-02-18

**Authors:** Hidemasa Kubo, Nobuki Yamaoka, Mizuki Tamai, Hajime Kamiya, Yosuke Kamada, Tomoyuki Nagata, Ken-ichiro Fukuda, Eigo Otsuji

**Affiliations:** 1 Department of Surgery, Kyoto Chubu Medical Center, 25 Yagi-Ueno, Yagi-cho, Nantan-shi, Kyoto, Japan; 2 Division of Digestive Surgery, Department of Surgery, Kyoto Prefectural University of Medicine, 465 Kajii-cho, Kamigyo-ku, Kyoto, Japan

**Keywords:** Polysplenia, Splenic torsion, Laparoscopic splenectomy

## Abstract

**Background:**

Polysplenia refers to the presence of two or more equal-sized spleens. Very rarely, one of the multiple spleens may develop torsion and infarction.

**Case presentation:**

A 21-year-old woman presented with left upper quadrant pain, the cause of which could not be diagnosed. She returned to our hospital, 2 days later, without any pain improvement. Enhanced computed tomography showed splenic infarction and polysplenia. Initially, we could not identify the cause of the infarction and started conservative therapy, which did not result in any improvement. Hence, we performed a splenectomy, after securing informed consent. Because the patient was a young woman, we opted for a laparoscopic approach. During surgery, we identified the cause of the infarction as spleen pedicle torsion; the infarcted spleen was excised using an automated suturing device. We completed the laparoscopic surgery without converting it to an open laparotomy, and the patient was discharged 4 days later. This was a rare case of polysplenia with splenic torsion.

**Conclusion:**

Laparoscopic splenectomy is minimally invasive and has cosmetic advantages. Thus, this approach may be considered as a treatment option for this condition.

## Background

In polysplenia, the bulk of the splenic tissue is reported to be divided into two or more equal masses and is often associated with various organ abnormalities [1]. This is different from an accessory spleen, which is a single, normal-sized spleen in the presence of one or more small, unequal masses of splenic tissue [1, 2]. In polysplenia, one of the multiple spleens may, very rarely, develop torsion and infarction. Hence, determining the diagnosis and a therapeutic strategy is difficult. We report the case of a 21-year-old woman with polysplenia who developed splenic torsion and infarction. She was successfully treated via laparoscopic splenectomy.

## Case presentation

**Fig. 1:**
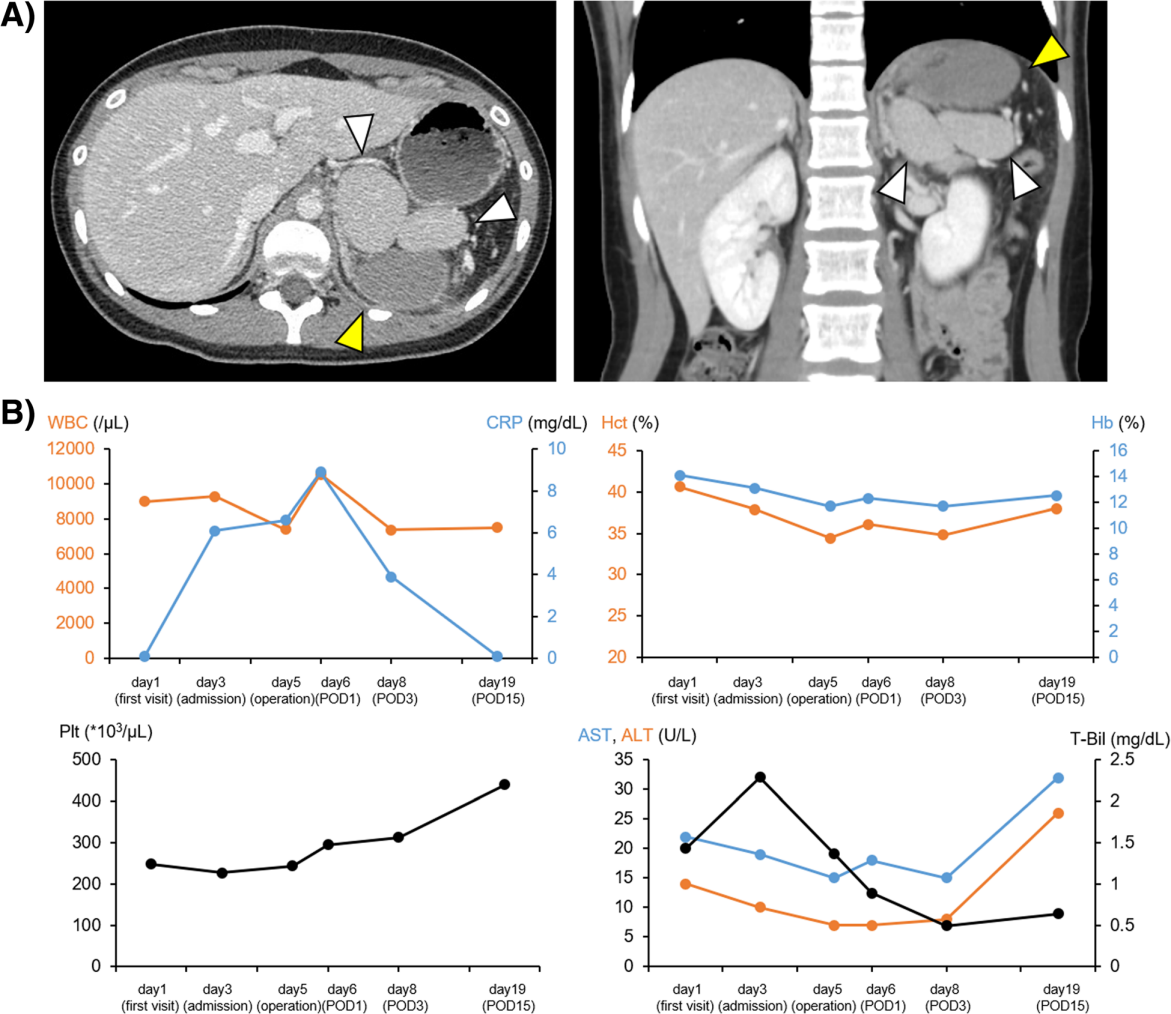
Findings upon admission. **a** Computed tomography shows the presence of three spleens, including one which is not enhanced. The yellow arrow head shows the infarcted spleen, and white arrow heads show the non-infarcted spleens. **b** Time course of white blood cell (WBC) count; and serum c-reactive protein (CRP), hemoglobin (Hb), hematocrit (Hct), platelet count (Plt), transaminases (AST, ALT), and total-bilirubin (T-Bil) levels. POD, postoperative day

**Fig. 2:**
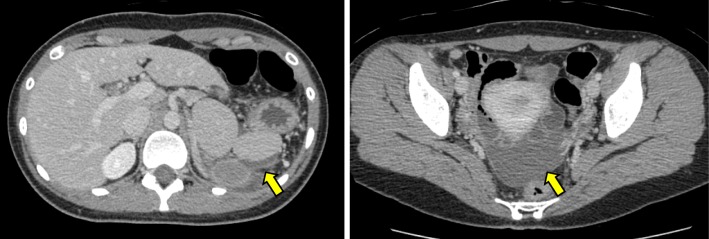
Computed tomography findings 2 days after admission. Ascites is apparent (yellow arrows)

A 21-year-old woman presented with left upper quadrant pain. She underwent routine blood tests and non-contrast computed tomography (CT). The blood tests did not show any abnormalities, and the CT showed the presence of three, similarly sized spleens, but no other abnormalities. As the patient did not have any other symptoms, she was sent home with a prescription for an analgesic. However, the abdominal pain did not improve and she returned to the hospital 2 days later. Her inflammatory markers were somewhat elevated, and an enhanced CT showed that one of the multiple spleens did not pick up the contrast (Fig. 1a). We diagnosed her with splenic infarction; however, the cause of the infarction was unclear, and torsion or embolism was considered possibilities. The patient was admitted and began conservative therapy, including fasting and antibiotic administration. However, neither her abdominal pain nor inflammatory marker levels improved (Fig. 1b). Hence, we performed a follow-up enhanced-CT scan, 2 days after admission, which showed that the splenic infarction had not improved and that ascitic fluid was present around the spleen and in the pelvic space (Fig. 2). At this point, we decided to surgically remove the infarcted spleen. Considering that the patient was a young woman, we elected to perform a laparoscopic splenectomy after receiving informed consent.

**Fig. 3:**
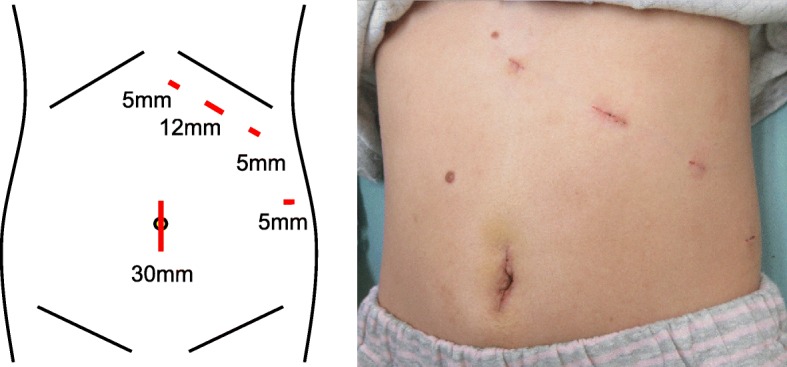
Port placement. The surgical incision schema is shown as is a postoperative picture depicting the incision sites

**Fig. 4:**
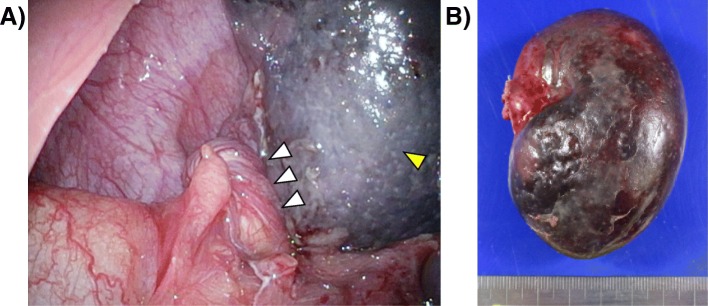
Intraoperative findings. **a** Yellow arrow head shows the infarcted spleen and white arrow heads show the twisted pedicle. **b** The resected spleen

The surgery was performed under general anesthesia, with the patient in a supine position and her legs spread apart. We created an umbilical incision and inserted three operating ports along the left subcostal margin (5 mm, 12 mm, and 5 mm in size), and a 5-mm operating port on the left side of the abdomen (Fig. 3). The port sites were selected along the lines of a left subcostal incision, in case conversion to open surgery became necessary. These port sites were also in a co-axial position to the surgeon. There were no adhesions observed in the abdominal cavity. First, we incised the omentum and opened the bursa, detecting two non-infarcted spleens in front of the pancreas. Behind these spleens, there was an infarcted spleen surrounded by fluid. We incised the inflamed adipose tissue around the spleen to expose the pedicle, which was twisted; consequently, we diagnosed splenic torsion (Fig. 4a). Using an automatic suturing device, we dissected the pedicle of the infarcted spleen. The umbilical incision was extended to remove the resected spleen (78 × 57 × 35 mm) (Fig. 4b). After confirming the absence of active bleeding, we sutured the incisions. The surgical time was 119 min, and there was little blood loss. The patient did not experience any complications and was discharged 4 days after surgery.

## Discussion

Polysplenia is a rare syndrome in which the patient has two or more equally sized spleens and various potential anomalies of the thoracic and abdominal organs [1]. Situs inversus is present in about 20% of patients and > 40% have cardiac anomalies [3]. Most patients with polysplenia are diagnosed during early childhood because of the various anomalies. In the present case, situs inversus was not evident in the CT scans, and echocardiography did not indicate any cardiac anomalies or functional disorders. Hence, this was the patient’s initial polysplenia diagnosis.

Diagnosing this condition is difficult because of its rarity. We suspected that the cause of the splenic infarction in this patient was torsion or an embolism. However, we also investigated the cause of her thrombophilia, including protein C and S activities, lupus anticoagulant, and anti-cardiolipin antibodies; all these parameters were within normal ranges (data not shown). The final diagnosis of splenic torsion was based on intraoperative findings.

Deciding on a therapeutic strategy for this condition was also difficult. Following the diagnosis of splenic infarction, we opted for conservative therapy under the assumption that the spleen had developed atrophic changes referring to a previous partial splenic embolization [4, 5]. Complications of embolization treatment are reported to include fever, abdominal pain, pneumonia, and splenic abscesses. In our case, the abdominal pain could not be controlled; hence, we decided upon surgical intervention. Surgery is thought to be the gold standard for a wandering spleen [6]. When a wandering spleen does not develop infarction or necrosis, splenopexy may be considered because of the risk of overwhelming post-splenectomy infection or thrombophilia. In our case, we decided to perform a splenectomy because the spleen was already infarcted and we believed that the effect of removing one of the three spleens would not significantly alter the patient’s splenic function. Postoperatively, however, the platelet count was slightly elevated (Fig. 1b). This elevation might have been a reflection of the change in splenic function after splenectomy.

Previous reports have described splenic torsion in conjunction with a wandering spleen [7–9] or an accessory spleen [10, 11]. However, few reports have described splenic torsion in the context of polysplenia. Based on our literature review, only five case reports of splenic torsion with polysplenia have been published in the English literature (Table 1) [1–3, 12, 13]. Only one case was managed conservatively [12]. Suthar et al. reported a similar condition but did not describe the cause of the infarction [14]. Applegate also reported a similar disease, but the case involved a single spleen with heterotaxy syndrome [15].

**Table 1:** Previous case reports of splenic torsion with polysplenia

Author	Year	Age	Sex	Total number of spleens (number of infarcted spleens)	Treatment
Ackerman et al. [12]	1982	7 months	F	3 (1)	Conservative
Griffiths et al. [1]	1984	23 years	F	2 (2)	Laparotomy
Lachmann et al. [13]	2006	9 years	F	No description (1)	Laparoscopy
Rasool et al. [3]	2011	2 days	F	7 (1)	Laparotomy
Dash et al. [2]	2013	12 years	M	5 (1)	Laparotomy
Present case		21 years	F	3 (1)	Laparoscopy

*M* male, *F* female

For our patient, we decided on a laparoscopic approach. Identifying the anatomy of the infarcted spleen’s pedicle based on the preoperative CT was difficult because the blood flow to the organ was interrupted. Intraoperatively, dissection of the pedicle was easier than during a normal splenectomy because the pedicle was not fixed and exfoliation was unnecessary. As a result, a successful laparoscopic splenectomy was completed, without needing to convert to an open laparotomy. The patient was discharged 4 days after surgery, without any complications. The laparoscopic surgery was also minimally invasive and provided the patient with cosmetic advantages.

## Conclusion

We reported a rare case of polysplenia with splenic torsion that was successfully treated via a laparoscopic splenectomy. Laparoscopic surgery is a useful treatment option for this condition.

## Abbreviation

CT: Computed tomography
